# Stakeholder engagement to introduce a standardised register for improved inpatient care of newborns and sick children in Bangladesh

**DOI:** 10.7189/jogh.14.04082

**Published:** 2024-05-17

**Authors:** Shafiqul Ameen, Sabit Saad Shafiq, Sabina Ashrafee, Ashfia Saberin, Palash Kumar Saha, Husam Md Shah Alam, Salmun Nahar, Supriya Sarkar, Sheikh Daud Adnan, Kabir ANM Ehtesham, Bushra Amena, Sabbir Ahmed, Md Nurul Khan, Goutom Banik, Sabrina Jabeen, Aniqa Tasnim Hossain, Sadman Sowmik Sarkar, Anisuddin Ahmed, Mohammod Jobayer Chisti, Muhammad Shariful Islam, Md Jahurul Islam, Shams El Arifeen, Ahmed Ehsanur Rahman

**Affiliations:** 1 International Centre for Diarrhoeal Disease Research, Bangladesh (icddr,b), Dhaka, Bangladesh; 2 Directorate General of Health Services (DGHS), Ministry of Health and Family Welfare, Dhaka, Bangladesh; 3 Save the Children, Dhaka Bangladesh; 4 UNICEF, Dhaka, Bangladesh; 5 Projahnmo Research Foundation, Dhaka, Bangladesh; 6 World Health Organization, Dhaka, Bangladesh; 7 Independent Consultant, Dhaka, Bangladesh

## Abstract

**Background:**

Despite a global decrease of 59% in under-five mortality rates from 1990 to 2021, child survival remains a pressing issue. This holds true for Bangladesh, as well. In response, the Government of Bangladesh introduced a standardised register for strengthening the inpatient management of newborns and sick children in 2021.

**Methods:**

We employed a comprehensive four-phase stakeholder engagement process to implement an inpatient register for newborns and sick children. The first stage included identifying and prioritising potential stakeholders at the national and district levels. We identified eight organisations involved in newborn and child health and selected 24 participants from various other sectors for workshops aimed at raising awareness about the register’s introduction. These stakeholders also participated in the register’s design, development strategies planning, and implementation phases. These phases were led by the ‘National Newborn Health and IMCI programme’ with support from various partners. A technical working group reviewed existing registers and helped prepare training materials. Feedback from each workshop was crucial in finalising the register.

**Results:**

The Government of Bangladesh has recognised the need for an indoor register for newborns and sick children, which was to be established in collaboration with development partners. This initiative can enhance the quality of care for sick children and increase service provider accountability. Due to its successful implementation, it will continue to be used in the Kushtia and Dinajpur districts, with plans for a nationwide scale-up. The Government has allocated funds in the next health sector programme for orientation and register printing. A strengths, weaknesses, opportunities, and threats (SWOT) analysis of the stakeholder engagement process highlighted strengths such as a context-specific approach and collaborative engagement, as well as challenges such as time resource requirements.

**Conclusions:**

Implementing an inpatient register for newborns and sick children through stakeholder engagement can effectively improve child health care services. Aside from challenges such as resource intensiveness and stakeholder commitments, success depended on the organising authority’s expertise in relationship building, budget allocation, time management, and workforce dedication. Therefore, strategic planning, staff recruitment, networking, and budgeting are crucial for successful stakeholder engagement and health care initiatives.

The global under-five mortality rate has significantly reduced by 59% from 1990 to 2021, decreasing from 93 to 38 deaths per 1000 live births. Despite this substantial achievement, the need for improvements in child survival remains critical. In 2021, an estimated 13 800 daily fatalities occurred among children under five, a significant portion of which were deemed to be preventable [1,2]. Similarly, Bangladesh has made considerable strides in reducing under-five mortality rates; according to the 2022 Bangladesh Health and Demographic Survey, it decreased from 48 to 31 deaths per 1000 live births between 2011 and 2022. Additionally, neonatal mortality rates significantly reduced from 27 per 1000 live births in 2017 to 20 in 2022 [3–6]. These advancements are attributed mainly to the country’s continuous efforts to enhance maternal and child health care services. However, child mortality remains a significant public health concern in Bangladesh. The primary causes of under-five mortality include complications from preterm birth, neonatal sepsis, childhood pneumonia, and diarrhoeal diseases. Approximately two-thirds of these fatalities occur within the first 28 days after birth [7,8].

To address this issue, the Ministry of Health in Bangladesh adopted the ‘Integrated Management of Childhood Illness’ (IMCI) advocated by the World Health Organization (WHO) for outpatient management of childhood illnesses [9,10], as well as the ‘WHO Pocket Book for Hospital Care of Children’ [11–13]. The Government of Bangladesh has also been supporting initiatives such as newborn care and assistance with newborn respiration in cases of birth asphyxia; administration of antenatal corticosteroids for suspected preterm labour; implementation of kangaroo mother care for preterm and low birth-weight infants; provision of injectable antibiotics for severe infections; and the establishment of special care newborn units for critically ill newborns [7,14,15].

Importantly, enhanced documentation practices can play a key role in disease monitoring and analysis of health care delivery patterns, thereby leading to the development of evidence-based care and service delivery models [16,17]. The implementation of a comprehensive health systems approach that ensures prompt delivery of high-quality inpatient care, identification of high-risk individuals, and systematic recording and reporting of clinical data could significantly reduce mortality rates and minimise morbidity [18–20]. The Government of Bangladesh has instituted service registers in various sectors related to the well-being of newborns and children, including postnatal care and IMCI corners; labour wards and operation theatres; special care newborn units; and kangaroo mother care facilities. These service registers compile monthly reports, which are subsequently submitted to a routine health information system (RHIS). However, paediatric inpatient departments currently do not have a dedicated service register or monthly reporting form.

Consequently, an implementation research study was launched in Bangladesh in 2021 to determine whether it would be possible to establish a standardised register for strengthening the inpatient management of newborns and sick children. The research was carried out by the National Newborn Health and IMCI programme (NNHP & IMCI) of the Directorate General of Health Services with assistance from the International Centre for Diarrhoeal Disease Research, Bangladesh (icddr,b) in the Kushtia and Dinajpur districts of Bangladesh. The aim was to integrate the suggested guidelines for implementing a standardised register for inpatient management of newborns and sick children into national policy and programme documents, as well as to evaluate the usability, acceptability, fidelity, and utility of this register using the implementation research guideline provided by the WHO [21,22].

Individuals, organisations, or societies that could potentially influence or be influenced by a project, research endeavour, or policy are called ‘stakeholders’ [23,24]. Stakeholder engagement, in turn, is a continuous and interactive process that aims to actively solicit the perspectives, experiences, and values of relevant and influential parties in agreeing on common objectives, while also serving to facilitate informed, clear, and efficient decision-making. This creates a two-way communication between the stakeholders and the researchers, which ultimately leads to informed choices regarding the prioritisation, implementation, and dissemination of the research outcomes [24,25]. At its cire, this requires involving a diverse group of stakeholders to enhance the research findings and acceptability [26,27]. Consequently, health research funding organisations increasingly emphasise stakeholder engagement as an approach which could increase the impact of research [28,29]

A recent study in Bangladesh on the integration of pulse oximeters in an IMCI package showed that involving stakeholders can positively impact national decision making [24]. In line with this, we wanted to incorporate key stakeholders at every stage of project design and development to ensure that the implementation process is guided by their decisions. It has also been shown that the adoption of context-specific leadership and ownership can enhance the scalability and sustainability of a project [27]. Through this analysis, we demonstrate the situation-specific engagement of a stakeholder method during programme development.

## METHODS

### Study sites

Based on the decision made by the NNHP & IMCI programme, we carried out this study in the Kushtia and Dinajpur districts of Bangladesh. While Kushtia district is located in the Khulna division of western Bangladesh and has a population exceeding two million, Dinajpur district lies in the Rangpur division of northern Bangladesh and is home to approximately three and a half million residents. Both districts are predominantly rural. They were selected based on the close alignment of under-five and neonatal mortality rates with national estimates [30]. The inpatient register was implemented across all district hospitals, as well as sub-district hospitals known as *upazila* health complexes (UHCs) in Kushtia and Dinajpur districts.

### Conceptual framework adoption

**Figure 1:**
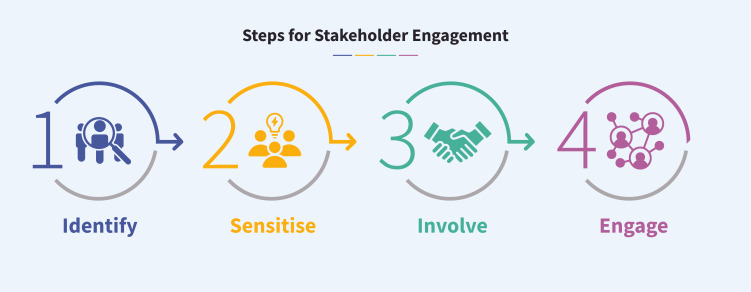
Adapted framework to implement inpatient register for newborns and sick children for stakeholder engagement in Bangladesh.

Stakeholder engagement in health research is driven by specific goals, opportunities, and contextual factors [24-26,31,32]. We have adapted a conceptual framework to guide our stakeholder engagement activities in our research (**Figure 1**). This framework consists of four key phases:

Phase A: Identifying and ranking stakeholders to prepare for subsequent phases of awareness raising, involvement, and engagement;
Phase B: Raising awareness among stakeholders about the background, rationale, significance, and approach of the planned research, with the intention of generating interest, confidence, and trust;
Phase C: Involving stakeholders in the planning process and reaching consensus on the implementation strategy;
Phase D: Engaging stakeholders at every stage from development to implementation.

### Phases of stakeholder engagement

#### Phase A: Identifying stakeholders for sensitisation, involvement, and engagement

The Ministry of Health and Family Welfare in Bangladesh is divided into the Directorate General of Family Planning and the Directorate General of Health Services, both of which are responsible for providing medical care, including child health services through dedicated child health programmes. The Government of Bangladesh collaborates with several development partners and professional bodies to operate and deliver child health services.

The NNHP & IMCI programme is working towards achieving the highest availability of health care services throughout the country for newborns and children. Our initial approach involved an exhaustive desk review to identify documents related to the register development process, as well as the conduct of interviews with nine key informants to identify the stakeholders at both national and district levels involved in the newborn health programme in Bangladesh (Tables S1 and S2 in **Online Supplementary Document****)**. This investigation led to the identification of eight organisations, encompassing government health programmes, professional societies, United Nations (UN) agencies, local and international non-governmental organisations, and health service providers.

**Figure 2:**
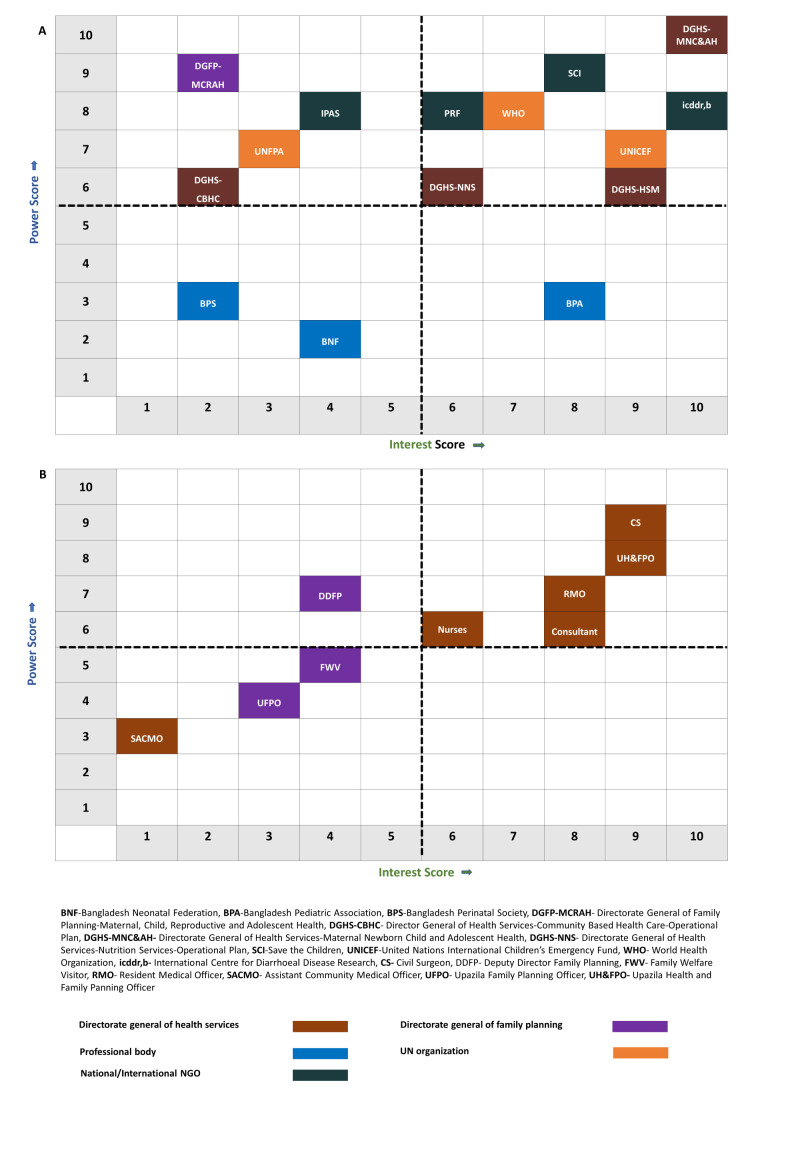
Stakeholder mapping involving both national and district level.

To optimise our limited resources, we arranged a consultative workshop with representatives from NNHP & IMCI programme, including the programme manager and their deputy programme managers. In this workshop, we employed the power-interest matrix as a tool to prioritise the stakeholder organisations for engagement [33–35]. First, workshop attendees used a 10-point Likert scale to rate each of the stakeholders based on their level of power and interest regarding involvement in the child health services in Bangladesh, with one indicating the lowest rating and ten signifying the highest rating. We then created the matrix itself (**Figure 2**) using the average of these scores. High-interest organisations had an average interest score of 6 or higher, while high-power organisations had an average power score of 6 or higher (Table S3 in **Online Supplementary Document**). At the national level, five organisations had substantial influence and interest, five had significant influence but limited interest, two had little influence but were very interested, and four had neither significant influence nor interest. It is recommended to notify all parties with significant influence or strong interest in the matter, with a particular emphasis on their active participation in following phases.

#### Phase B: Raising awareness among stakeholders for understanding

We conducted four workshops with 24 participants from various organisations, including national entities and development partners such as the NNHP & IMCI programme and the Hospital Services Management programme of the Directorate General of Health Services; the National Nutrition Services of the Institute of Public Health & Nutrition; the icddr,b; the United Nations Children’s Fund (UNICEF); the WHO; and Save the Children (SCI). The workshops were led by the programme manager of the NNHP & IMCI programme, who thereby symbolised government leadership. The primary aim was to scrutinise the operational aspects of introducing inpatient register for newborns and sick children in Bangladesh. Two additional workshops were held in the Kushtia and Dinajpur districts to incorporate local stakeholders. The attendees included the civil surgeon (district health manager), superintendent (head of the district hospital), *upazila* health and family planning officer (sub-district health manager), facility paediatric consultants, senior staff nurses, and representatives from the NNHP & IMCI programme. During these workshops, stakeholders provided valuable insights on how to successfully roll out this initiative in the chosen districts.

#### Phase C: Involving stakeholders in the planning process

During this phase, we arranged a workshop with significant interest and strong power stakeholders to discuss the development of the inpatient register. The PM of NNHP & IMCI programme led the workshop and initiated the discussion on developing a strategic plan for introducing a standard inpatient register along with the developing partners from WHO, SCI, UNICEF, and icddr,b. All the participants agreed on the process of implementing the register, while the NNHP & IMCI programme decided to include it in the paediatric inpatient setting to improve the quality of care. During this workshop, development partners also reached a consensus that they will provide the register in the initial stage of the implementation in some of the selected facilities.

From each of the selected developing partners, we designed a working group with experts in child health to implement and monitor the progress of the register in selected facilities. Technical experts from the working group suggested assessing the feasibility of the inpatient register. Therefore, icddr,b took the lead to examine the feasibility in Kushtia and Dinajpur districts. We held an additional workshop to identify research questions and specific objectives.

Two consultative workshops were held in Kushtia and Dinajpur districts to finalise the implementation plan. The workshops were led by the district health managers (civil surgeons) and attended by the sub-district health managers, paediatric consultants, medical officers, and nurses. It was decided that all UHCs that have inpatient services should be included. Consequently, five UHCs from Kushtia district and all 12 UHCs from Dinajpur district were included. However, for implementation research purposes, only the Kushtia district hospital and Kumarkhali UHC were selected from Kushtia district, and Dinajpur district hospital and Hakimpur UHC were selected from Dinajpur district by the NNHP & IMCI programme during the workshops.

#### Phase D: Engaging stakeholders at every stage from development to implementation

Phase D was a collaborative effort between NNHP & IMCI programme, icddr,b, and other stakeholders to develop and implement a register in selected facilities to assess feasibility. The process involved the formation of a technical working group consisting of 24 child health experts from influential groups, who reviewed existing documents and registers used to evaluate care quality across various sectors (Table S4 in **Online Supplementary Document**). The technical working group initiated the discussion on developing an inpatient register after a thorough review of existing registers for newborn and child health, while icddr,b provided continuous technical support throughout this process. The group also proposed a monthly reporting form and a training manual in both English and Bangla to guide nurses. Meanwhile, the NNHP & IMCI programme organised Training of Trainers workshops with assistance from development partners to familiarise participants with the register and prepare them to train nurses at their respective facilities. Afterwards, a series of training workshops were arranged at each selected facility to train 176 paediatric indoor staff nurses from the Kushtia and Dinajpur districts. Feedback on the register and monthly reporting form was collected during these interactive sessions.

This comprehensive approach ensured that all stakeholders were actively involved in the process, thereby enhancing the feasibility and effectiveness of the register implementation.

#### Major activities of the stakeholder engagement process

Inpatient register finalisation: after the training of master trainers and paediatric indoor service providers, the working group members deliberated on the feedback received and updated the register and monthly reporting form. The final register, endorsed by the programme manager of the NNHP & IMCI programme, is divided into two sections with 14 variables (**Box 1**).

Box 1 **Variables of the inpatient register**

**Columns to be filled up at the time of admission:**

Column 1: Serial number

Column 2: Registration number

Column 3: Date and time of admission

Column 4: The place where the child first came in this facility

Column 5: Child's identity, address and parent's mobile phone number

Column 6: Information on referral in this facility

Column 7: On admission examination

Column 8: Danger sign present at admission

**Columns to be filled up at the time of discharge:**

Column 9: Investigation done

Column 10: Care received during admission

Column 11: Drugs received during admission

Column 12: Final diagnosis with ICD 10 code during discharge

Column 13: Outcome of treatment

Column 14: Remarks and signature

Implementation research and involving the stakeholders: to assess the register’s feasibility, an implementation research plan was designed and developed, with contributions from icddr,b, the NNHP & IMCI programme, and other development partners.

District and sub-district level participation during the research: involvement at the district and sub-district levels was crucial during the research phase. Advisory committees were established in both Kushtia and Dinajpur districts to provide guidance during this phase. The committees included additional members such as sub-district managers, paediatric consultants, medical officers and nurses. A timeline was set for the data collection process, with all committee members assisting the icddr,b team throughout the implementation research.

### Resources required for stakeholder engagement

**Figure 3:**
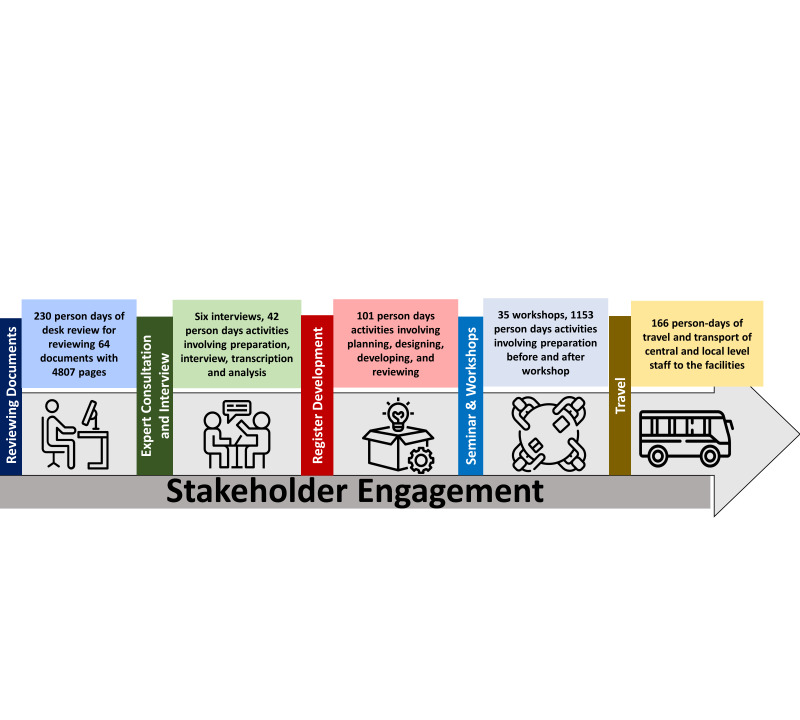
Resources used and focussed on stakeholder collaboration initiatives.

An overview of the stakeholder engagement activities and resource needed illustrated in **Figure 3**.

#### Document review process

Significant effort was put into reviewing important documents. This task took 230-person days to prepare the necessary list and involved going through 64 different documents comprising 4807 pages (Table S1 in **Online Supplementary Document**).

#### Expert consultation and interviews

Six newborn and child health experts from the NNHP & IMCI programme and three experts from icddr,b were consulted to gain insights for stakeholder engagement. The interview process required nine hours, with an additional 22 person-hours needed for transcription and analysis.

#### Register development

The comprehensive development of registers demanded 810 person-hours of diligent work, equivalent to 101 person-days (assuming an eight-hour workday). This time was spent on meticulous planning, designing, developing, and reviewing.

#### Seminars and workshops

The stakeholder engagement process necessitated 1154 person-days dedicated to workshops and seminars. Thirteen central workshops required approximately 292 person-days of active participation from newborn and child health experts. Additionally, district-level involvement necessitated 16 person-days for eight meetings.

#### Travel

Engaging the stakeholders required extensive travel amounting to 166 person-days. Central staff members needed to travel for 54 days, while field staff required 97 days of travel to implement the register in the facilities.

### Milestones

**Figure 4:**
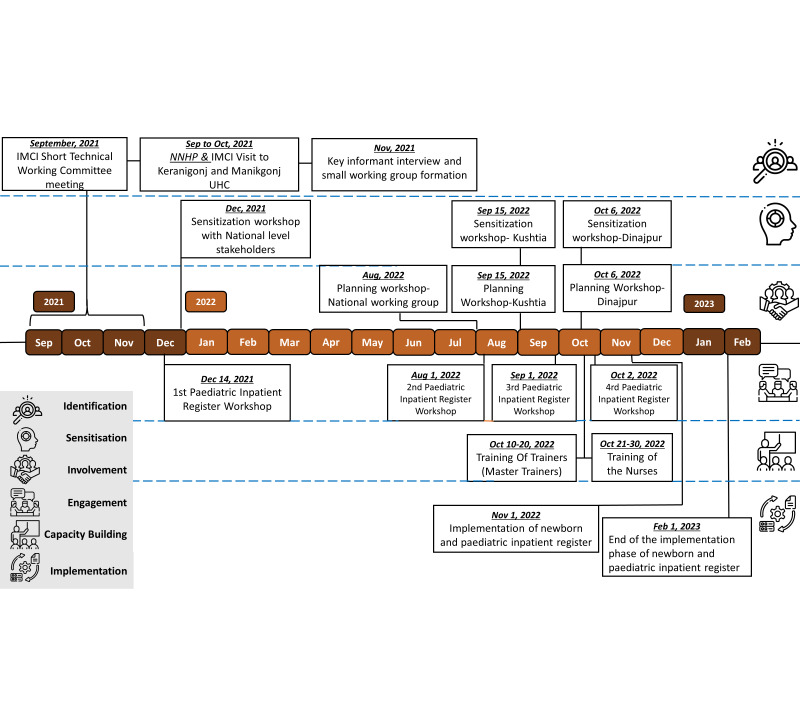
Stakeholder engagement key timeline.

**Figure 4** presents an overview of the timeline of the key stakeholder engagement activities.

Phase A – Identification (September to November 2021): This initial phase involved the formation of a working group.

Phase B – Sensitisation (December 2021, September 2022, October 2022): The sensitisation process commenced in December 2021 with national-level stakeholders. This was followed by two additional sensitisation sessions with potential stakeholders held in the Kushtia district in September 2022 and the Dinajpur district in October 2022.

Phase C – Involvement (August 2022 to October 2022): Stakeholders were actively involved in the planning, designing, and development of the register. Three planning workshops were conducted during this period; one was held at the national level, while the other two were conducted in the Kushtia and Dinajpur districts.

Phase D – Engagement (December 2021 to October 2022): This phase saw a series of four stakeholder engagement workshops. These workshops, conducted between December 2021 and October 2022, facilitated a variety of discussions.

### Ethical considerations

We obtained ethical approval (PR-21112) from the Institutional Review Board of icddr,b, as well as administrative approvals from the NNHP & IMCI programme to conduct the study in Kushtia and Dinajpur districts.

## RESULTS

### Stakeholder engagement and its impact

#### Acknowledgement from the Government of Bangladesh

Through the NNHP & IMCI programme, the Government of Bangladesh and other development partners have recognised the need for a standard inpatient register for newborns and sick children to enhance the quality of care. They are confident that this register will not only improve indoor management for children aged 0–59 months, but also increase the accountability of service providers in delivering proper management within resource constraints.

#### Future sustainability and inclusion in next sector programme

Looking ahead, the NNHP & IMCI programme has decided to continue using the inpatient register in both the Kushtia and the Dinajpur district, with support from icddr,b. They are also engaged in ongoing discussions with the management information system of the Directorate General of Health Services to incorporate necessary indicators into the RHIS. A significant decision made post-implementation is the inclusion of the inpatient register in the next health sector programme. This implies that the Government of Bangladesh is planning to scale up the use of the register nationwide. To facilitate this, funds have been allocated for orientation workshops and printing of registers.

## DISCUSSION

### Lessons learned

**Figure 5:**
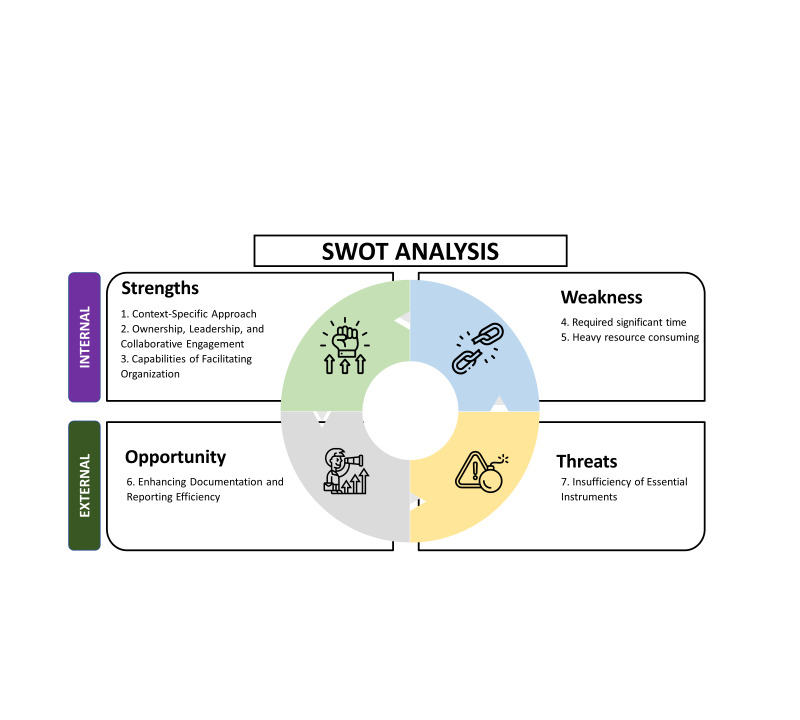
SWOT analysis.

The stakeholder engagement process was analysed using a strength, weakness, opportunity, and threat (SWOT) analysis [36,37]. The key takeaways are highlighted in **Figure 5**.

### Strengths

#### Context-specific approach

The research aimed to strengthen current policy through the implementation of a standard inpatient register for newborns and sick children. Its findings could help bridge the identified gap in the quality of child health care. A key stakeholder engagement framework was adopted to engage key interest groups with significant influence on the policy. This framework guided the design, development, and implementation of the entire process and optimised the utilisation of scarce resources. Therefore, this stakeholder engagement framework can be emphasised for use according to specific goals and contexts.

#### Ownership, leadership, and collaborative engagement

The NNHP & IMCI programme of the Government of Bangladesh demonstrated ownership and leadership. They led the establishment and oversight of the national working group from the beginning to the end of the implementation process. They also demonstrated leadership in the workshops, in preparing the implementation plan, and in collaborating with other development partners. This led to all participating partners reaching a consensus on introducing a standard register for newborns and sick children.

#### Capabilities of facilitating organisation

icddr,b demonstrated their expertise in implementing the register for newborns and sick children through their connections with other influential stakeholders involved in child health. They played a pivotal role in communication and organisation throughout the stakeholder engagement process due to their extensive technical expertise in this area. Therefore, understanding the capabilities and role of the implementing organisation is imperative before deciding on an approach to engage stakeholders.

### Weakness

#### Required significant time

The entire process took one and a half years to achieve the desired policy implications of implementing the register. Coordinating with high-influence, high-power stakeholders for workshops was challenging due to their busy schedules. Furthermore, extensive preparation was required for these workshops, including comprehensive desk reviews that took weeks to due to the need to compile all necessary documents. Consequently, the facilitating organisation had to invest a significant amount of time to effectively arrange and engage stakeholders.

#### Highly resource-intensive

Identifying potential stakeholders and reviewing documents for register build-up required considerable time and travel for the staff involved in the project. Organising workshops at both the national and district levels necessitated extensive travel. This highlight that budget allocation is a crucial aspect of the stakeholder engagement process.

### Opportunity

#### Enhancing documentation and reporting efficiency

Bangladesh is currently facing a problem with the absence of a standardised and structured inpatient register for newborns and sick children, leading to inconsistent data quality. The introduction of an inpatient register for newborns and sick children presents a significant opportunity for the Government of Bangladesh. This initiative ensures uniform documentation of diagnoses and treatment procedures, thereby enhancing the continuity of care and guaranteeing the precision and consistency of information in monthly reports. Consequently, health care professionals can generate monthly reports with greater efficiency, reducing the time spent on locating individual records and augmenting overall patient care coordination.

### Threats

#### Insufficiency of essential instruments

The lack of crucial medical instruments such as pulse oximeters, the mid-upper arm circumference tape, and height and weight measurement machines pose a significant threat to data collection and patient care. The scarcity of resources can obstruct proper record-keeping and potentially influence patient outcomes adversely.

## CONCLUSIONS

In this paper, we explain the significance of stakeholder engagement when executed effectively. Our process encourages top-tier health policymakers to contemplate the initiation of registers in indoor environments, a move that could potentially improve the quality of child health care services. It also highlights that stakeholder-backed decisions are more likely to be universally accepted and to become scalable.

However, the process is not devoid of challenges. Stakeholder engagement is a resource-intensive activity that requires considerable time and meticulous planning for the successful design and development of an accepted implementation plan. The feasibility of this is often compromised due to the stakeholders' heavy workload and other commitments.

Moreover, the efficacy of this approach is heavily reliant on the facilitating organisation's proficiency and experience in fostering relationships and networks with stakeholders. Proper budget allocation, efficient time management, and a dedicated workforce can fast-track the success of this process. Hence, planning, staff recruitment, time management, networking, and budgeting are key to a successful stakeholder engagement process.

## Additional material


Online Supplementary Document

## References

[1] United Nations Children’s Fund. Under-five mortality. 2023. Available: https://data.unicef.org/topic/child-survival/under-five-mortality/. Accessed 24 April 2024.

[2] World Health Organization. Child mortality and causes of death. 2023. Available: https://www.who.int/data/gho/data/themes/topics/topic-details/GHO/child-mortality-and-causes-of-death#:~:text=Since%201990%2C%20the%20global%20under,to%202.3%20million%20in%202021. Accessed: 24 April 2024.

[3] National Institute of Population Research and Training (NIPORT), Mitra and Associates, ICF International. Bangladesh Demographic and Health Survey 2011. Dhaka, Bangladesh and Calverton, Maryland, USA: NIPORT, Mitra and Associates, and ICF International; 2013. Available: https://dhsprogram.com/pubs/pdf/FR265/FR265.pdf. Accessed: 24 April 2024.

[4] National Institute of Population Research and Training (NIPORT), ICF. Bangladesh Demographic and Health Survey 2017-18. Dhaka, Bangladesh, and Rockville, Maryland, USA: NIPORT and ICF; 2020. Available: https://dhsprogram.com/pubs/pdf/FR344/FR344.pdf. Accessed: 24 April 2024.

[5] National Institute of Population Research and Training (NIPORT), ICF. Bangladesh Demographic and Health Survey 2022: Key Indicators Report. Dhaka, Bangladesh, and Rockville, Maryland, USA: NIPORT and ICF; 2023. Available: https://dhsprogram.com/pubs/pdf/PR148/PR148.pdf. Accessed: 24 April 2024.

[6] National Institute of Population Research and Training (NIPORT), Mitra and Associates, ICF International. Bangladesh Demographic and Health Survey 2014. Dhaka, Bangladesh, and Rockville, Maryland, USA: NIPORT, Mitra and Associates, and ICF International; 2016. Available: https://dhsprogram.com/pubs/pdf/FR311/FR311.pdf. Accessed: 24 April 2024.

[7] Rahman AE Hossain AT Siddique AB Jabeen S Chisti MJ Dockrell DH Child mortality in Bangladesh – why, when, where and how? A national survey-based analysis. J Glob Health. 2021;11:04052. 10.7189/jogh.11.04052 34552721 PMC8442576

[8] Binagwaho A, Udoh K, Ntawukuriryayo T, Faruk MO, Mahmood HR, Huda FA, et al. Exemplars in Under-5 Mortality: Bangladesh Case Study. 2019. Kigali, Rwanda and Dhaka, Bangladesh: University of Global Health Equity, icddr,b; 2019. Available: https://www.exemplars.health/-/media/files/egh/resources/underfive-mortality/bangladesh/bangladesh-case-study-_-final-28082020.pdf. Accessed: 24 April 2024.

[9] World Health Organization. Integrated management of childhood illness – chart booklet. Geneva, Switzerland: World Health Organization; 2014. Available: https://www.who.int/publications/m/item/integrated-management-of-childhood-illness—chart-booklet-(march-2014). Accessed: 24 April 2024.

[10] Carai S Kuttumuratova A Boderscova L Khachatryan H Lejnev I Monolbaev K Review of Integrated Management of Childhood Illness (IMCI) in 16 countries in Central Asia and Europe: implications for primary healthcare in the era of universal health coverage. Arch Dis Child. 2019;104:1143–9. 10.1136/archdischild-2019-317072 31558445 PMC6900244

[11] World Health Organization. Pocket book of hospital care for children: guidelines for the management of common childhood illnesses. Geneva, Switzerland: World Health Organization; 2013. Available: https://www.ncbi.nlm.nih.gov/books/NBK154447/. Accessed: 24 April 2024. 24006557

[12] Ashraf H Alam NH Sultana M Jahan SA Begum N Farzana S Day clinic vs. hospital care of pneumonia and severe malnutrition in children under five: a randomised trial. Trop Med Int Health. 2019;24:922–31. 10.1111/tmi.13242 31046165

[13] Chisti MJ Salam MA Bardhan PK Faruque AS Shahid AS Shahunja KM Severe Sepsis in Severely Malnourished Young Bangladeshi Children with Pneumonia: A Retrospective Case Control Study. PLoS One. 2015;10:e0139966. 10.1371/journal.pone.0139966 26440279 PMC4595075

[14] Nahar T Azad K Aumon BH Younes L Shaha S Kuddus A Scaling up community mobilisation through women’s groups for maternal and neonatal health: experiences from rural Bangladesh. BMC Pregnancy Childbirth. 2012;12:5. 10.1186/1471-2393-12-5 22273440 PMC3298477

[15] Rahman AE Herrera S Rubayet S Banik G Hasan R Ahsan Z Managing possible serious bacterial infection of young infants where referral is not possible: Lessons from the early implementation experience in Kushtia District learning laboratory, Bangladesh. PLoS One. 2020;15:e0232675. 10.1371/journal.pone.0232675 32392209 PMC7213695

[16] Gliklich RE, Dreyer NA, Leavy MB, editors. Registries for Evaluating Patient Outcomes: A User's Guide. 3rd ed. Rockville, Maryland: Agency for Healthcare Research and Quality; 2014. Available: https://www.ncbi.nlm.nih.gov/books/NBK208616/. Accessed: 24 April 2024. 24945055

[17] Madewell ZJ Whitney CG Velaphi S Mutevedzi P Mahtab S Madhi SA Prioritizing Health Care Strategies to Reduce Childhood Mortality. JAMA Netw Open. 2022;5:e2237689. 10.1001/jamanetworkopen.2022.37689 36269354 PMC9587481

[18] Duggan AK Starfield B DeAngelis C Structured encounter form: the impact on provider performance and recording of well-child care. Pediatrics. 1990;85:104–13. 10.1542/peds.85.1.104 2296477

[19] Madhok R Crossing the quality chasm: lessons from health care quality improvement efforts in England. Proc (Bayl Univ Med Cent). 2002;15:77–83. 10.1080/08998280.2002.11927816 16333409 PMC1276338

[20] World Health Organization, United Nations Children’s Fund. Every newborn: an action plan to end preventable deaths. Geneva, Switzerland: World Health Organization; 2014. Available: https://www.who.int/publications-detail-redirect/9789241507448. Accessed: 24 April 2024.

[21] Peters DH, Tran NT, Adam T. Implementation research in health: a practical guide. Geneva, Switzerland: World Health Organization; 2013. Available: https://ahpsr.who.int/publications/i/item/9789241506212. Accessed: 24 April 2024.

[22] Proctor E Silmere H Raghavan R Hovmand P Aarons G Bunger A Outcomes for implementation research: conceptual distinctions, measurement challenges, and research agenda. Adm Policy Ment Health. 2011;38:65–76. 10.1007/s10488-010-0319-7 20957426 PMC3068522

[23] Deverka PA Lavallee DC Desai PJ Esmail LC Ramsey SD Veenstra DL Stakeholder participation in comparative effectiveness research: defining a framework for effective engagement. J Comp Eff Res. 2012;1:181–94. 10.2217/cer.12.7 22707880 PMC3371639

[24] Rahman AE Jabeen S Fernandes G Banik G Islam J Ameen S Introducing pulse oximetry in routine IMCI services in Bangladesh: a context-driven approach to influence policy and programme through stakeholder engagement. J Glob Health. 2022;12:06001. 10.7189/jogh.12.06001 35441007 PMC8994831

[25] Concannon TW Fuster M Saunders T Patel K Wong JB Leslie LK A systematic review of stakeholder engagement in comparative effectiveness and patient-centered outcomes research. J Gen Intern Med. 2014;29:1692–701. 10.1007/s11606-014-2878-x 24893581 PMC4242886

[26] Boaz A Hanney S Borst R O’Shea A Kok M How to engage stakeholders in research: design principles to support improvement. Health Res Policy Syst. 2018;16:60. 10.1186/s12961-018-0337-6 29996848 PMC6042393

[27] Huzzard T Achieving impact: Exploring the challenge of stakeholder engagement. Eur J Work Organ Psychol. 2021;30:379–89. 10.1080/1359432X.2020.1761875

[28] Prebanić KR Vukomanović M Exploring Stakeholder Engagement Process as the Success Factor for Infrastructure Projects. Buildings. 2023;13:1785. 10.3390/buildings13071785

[29] Kok MO Gyapong JO Wolffers I Ofori-Adjei D Ruitenberg J Which health research gets used and why? An empirical analysis of 30 cases. Health Res Policy Syst. 2016;14:36. 10.1186/s12961-016-0107-2 27188305 PMC4869365

[30] National Institute of Population Research and Training (NIPORT), International Centre for Diarrhoeal Disease Research, Bangladesh (icddr,b), MEASURE Evaluation. Bangladesh District Level Socio-demographic and Health Care Utilization Indicators. Dhaka, Bangladesh, and Chapel Hill, North Carolina, USA: NIPORT, icddr,b, and MEASURE Evaluation; 2019. Available: https://www.measureevaluation.org/resources/publications/tr-20-395.html. Accessed: 24 April 2024.

[31] Concannon TW Grant S Welch V Petkovic J Selby J Crowe S Practical guidance for involving stakeholders in health research. J Gen Intern Med. 2019;34:458–63. 10.1007/s11606-018-4738-6 30565151 PMC6420667

[32] Mallery C Ganachari D Smeeding J Fernandez J Lavalle D Siegel J Innovative Methods for Stakeholder Engagement: An Environmental Scan. Value Health. 2012;15:A14. 10.1016/j.jval.2012.03.082

[33] Joshi A Kale S Chandel S Pal DK Likert scale: Explored and explained. Br J Appl Sci Technol. 2015;7:396–403. 10.9734/BJAST/2015/14975

[34] Slabá M. Stakeholder power-interest matrix and stakeholder-responsibility matrix in corporate social responsibility. In: Löster T, Pavelka T, editors. The 8th International Days of Statistics and Economics – Conference Proceedings; 2014 Sep 11-13; Prague, Czech Republic. Prague University of Economics and Business: Prague; 2014. p. 1366-74.

[35] Pacagnella Júnior AC Porto GS Pacífico O Salgado Júnior AP Project Stakeholder Management: A Case Study of a Brazilian Science Park. J Technol Manag Innov. 2015;10:39–49. 10.4067/S0718-27242015000200004

[36] Helms MM Nixon J Exploring SWOT analysis – where are we now? Journal of Strategy and Management. 2010;3:215–51. 10.1108/17554251011064837

[37] Namugenyi C Nimmagadda SL Reiners T Design of a SWOT Analysis Model and its Evaluation in Diverse Digital Business Ecosystem Contexts. Procedia Comput Sci. 2019;159:1145–54. 10.1016/j.procs.2019.09.283

