# Supplementary material for: Stakeholder engagement to introduce a standardised register for improved inpatient care of newborns and sick children in Bangladesh
Source: J Glob Health. 2024 May 17;14:04082. doi: 10.7189/jogh.14.04082 (PMC11096775; doi:10.7189/jogh.14.04082)
Supplement: Online Supplementary Document [file jogh-14-04082-s001.pdf]

Online Supplementary Document:

Table S1: List of documents reviewed during development of inpatient register of newborn and sick children

Sl no	Name of the documents	Number of pages
1.	IMCI chart booklet WHO 2014	80
2.	IMNCI Sick Young Infant chart booklet WHO 2019	36
3.	Bangladesh adopted IMCI chart booklet 2018	43
4.	IMCI training manual - 6. Management of young infants aged up to 2 months	70
5.	WHO pocketbook	438
6.	UNICEF WHO, World Bank, United Nations. Levels and trends in child mortality: report 2017. 2017.	40
7.	Lawn JE, Blencowe H, Oza S, You D, Lee ACC, Waiswa P, et al. Every Newborn: progress, priorities, and potential beyond survival. The Lancet. 2014;384(9938):189-205.	16
8.	Lee AC, Katz J, Blencowe H, Cousens S, Kozuki N, Vogel JP, et al. National and regional estimates of term and preterm babies born small for gestational age in 138 low-income and middle-income countries in 2010. (2214-109X (Electronic)).	11
9.	Katz J, Lee AC, Kozuki N, Lawn JE, Cousens S, Blencowe H, et al. Mortality risk in preterm and small-for-gestational-age infants in low-income and middle-income countries: a pooled country analysis. (1474-547X (Electronic)).	9
10.	Blencowe H, Cousens S Fau - Oestergaard MZ, Oestergaard Mz Fau - Chou D, Chou D Fau - Moller A-B, Moller Ab Fau - Narwal R, Narwal R Fau - Adler A, et al. National, regional, and worldwide estimates of preterm birth rates in the year 2010 with time trends since 1990 for selected countries: a systematic analysis and implications. (1474-547X (Electronic)).	11
11.	The Pertnership for Maternal N, and Child Health. Newborn death and illness 2011 [cited 2021 August 11]. Available from: https://www.who.int/pmnch/media/press_materials/fs/fs_newborndealth_illness/en/.	60
12.	Wardlaw TM, Johansson EW, Hodge MJ. Pneumonia: the forgotten killer of children: Unicef; 2006.	44
13.	National Institute of Population R, Training U. Bangladesh Demographic and Health Survey 2017-18. 2019.	92
14.	Bhutta ZA, Das JK, Bahl R, Lawn JE, Salam RA, Paul VK, et al. Can available interventions end preventable deaths in mothers, newborn babies, and stillbirths, and at what cost? The Lancet. 2014;384(9940):347-70.	24
15.	Lawn JE, Kinney MV, Black RE, Pitt C, Cousens S, Kerber K, et al. Newborn survival: a multi-country analysis of a decade of change. Health policy and planning. 2012;27(suppl_3):iii6-iii28.	23
16.	Duggan AK, Starfield B Fau - DeAngelis C, DeAngelis C. Structured encounter form: the impact on provider performance and recording of well-child care. (0031-4005 (Print)).	1
17.	Mwakyusa S, Wamae A, Wasunna A, Were F, Esamai F, Ogutu B, et al. Implementation of a structured paediatric admission record for district hospitals in Kenya – results of a pilot study. BMC International Health and Human Rights. 2006;6(1).	7

Sl no	Name of the documents	Number of pages
18.	UNICEF W. Every Newborn: an action plan to end preventable deaths. UNICEF, WHO; 2014.	58
19.	Moxon SG, Lawn JE, Dickson KE, Simen-Kapeu A, Gupta G, Deorari A, et al. Inpatient care of small and sick newborns: a multi-country analysis of health system bottlenecks and potential solutions. BMC pregnancy and childbirth. 2015;15(2):S7.	19
20.	Lawn JE, Davidge R, Paul VK, von Xylander S, de Graft Johnson J, Costello A, et al. Born too soon: care for the preterm baby. Reproductive health. 2013;10 Suppl 1(Suppl 1):S5-S.	19
21.	Blencowe H, Lawn JE, Vazquez T, Fielder A, Gilbert C. Preterm-associated visual impairment and estimates of retinopathy of prematurity at regional and global levels for 2010. Pediatric research. 2013;74 Suppl 1(Suppl 1):35-49.	16
22.	Wall SN, Lee ACC, Niermeyer S, English M, Keenan WJ, Carlo W, et al. Neonatal resuscitation in low-resource settings: what, who, and how to overcome challenges to scale up? International journal of gynaecology and obstetrics: the official organ of the International Federation of Gynaecology and Obstetrics. 2009;107 Suppl 1(Suppl 1):S47-S64.	36
23.	Enweronu-Laryea C, Dickson KE, Moxon SG, Simen-Kapeu A, Nyange C, Niermeyer S, et al. Basic newborn care and neonatal resuscitation: a multi-country analysis of health system bottlenecks and potential solutions. BMC pregnancy and childbirth. 2015;15 Suppl 2(Suppl 2):S4-S.	20
24.	Rahman AE, Hossain AT, Zaman SB, Salim N, K.C A, Day LT, et al. Antibiotic use for inpatient newborn care with suspected infection: EN-BIRTH multi-country validation study. BMC Pregnancy Childbirth. 2021;21(1):229.	18
25.	Finkelstein J, Cha EM. Using a Mobile App to Promote Smoking Cessation in Hospitalized Patients. JMIR Mhealth Uhealth. 2016;4(2):e59.	16
26.	English LA-O, Dunsmuir D Auid- Orcid: --- Fau - Kumbakumba E, Kumbakumba E Auid- Orcid: --- Fau - Ansermino JM, Ansermino Jm Auid- Orcid: --- Fau - Larson CP, Larson Cp Auid- Orcid: --- Fau - Lester R, Lester R Auid- Orcid: --- Fau - Barigye C, et al. The PAediatric Risk Assessment (PARA) Mobile App to Reduce Postdischarge Child Mortality: Design, Usability, and Feasibility for Health Care Workers in Uganda. (2291-5222 (Print)).	11
27.	Kushniruk AW, Patel Vl Fau - Cimino JJ, Cimino JJ. Usability testing in medical informatics: cognitive approaches to evaluation of information systems and user interfaces. (1091-8280 (Print)).	06
28.	Ng AW, Lo H, Chan A, editors. Measuring the Usability of Safety Signs: A use of system usability scale (SUS). proceedings of the International MultiConference of Engineers and Computer Scientists; 2011: Citeseer.	07
29.	Brooke J. System Usability Scale (SUS): A quick and dirty usability scale. 1996.	08
30.	Davis FD. A technology acceptance model for empirically testing new end-user information systems: Theory and results: Massachusetts Institute of Technology; 1985.	292
31.	Davis FDJMq. Perceived usefulness, perceived ease of use, and user acceptance of information technology. 1989:319-40.	24
32.	Gillenson ML, Sherrell DLJI, management. Enticing online consumers: an extended technology acceptance perspective. 2002;39(8):705-19.	15

Sl no	Name of the documents	Number of pages
33.	National Institute of Population Research and Training (NIPORT), Mitra and Associates, and ICF International. Bangladesh Demographic and Health Survey 2014.	354
34.	Clark S. Son preference and sex composition of children: Evidence from India. Demography. 2000;37(1):95-108.	15
35.	UNICEF. The state of the world's children 2007: women and children: the double dividend of gender equality: Unicef; 2006.	160
36.	UNICEF. UNICEF Annual Report. 2012.	52
37.	National Institute of Population Research and Training (NIPORT) MaA, and ICF International,. Bangladesh Demographic and Health Survey 2011. 2011.	458
38.	Ahmed S, Sobhan F, Islam A. Neonatal Morbidity and Care-seeking Behaviour in Rural Bangladesh. Journal of Tropical Pediatrics. 2001;47(2):98-105.	8
39.	Rosenstock S, Katz J, Mullany LC, Khatry SK, LeClerq SC, Darmstadt GL, et al. Sex differences in morbidity and care-seeking during the neonatal period in rural southern Nepal. Journal of Health, Population and Nutrition. 2015;33(1):11.	11
40.	Kerber KJ, de Graft-Johnson JE, Bhutta ZA, Okong P, Starrs A, Lawn JE. Continuum of care for maternal, newborn, and child health: from slogan to service delivery. The Lancet. 2007;370(9595):1358-69.	12
41.	Roy SK, Jolly SP, Shafique S, Fuchs GJ, Mahmud Z, Chakraborty B, et al. Prevention of malnutrition among young children in rural Bangladesh by a food-health-care educational intervention: a randomized, controlled trial. Food and nutrition bulletin. 2007;28(4):375-83.	10
42.	Joshi S. Female household-headship in rural Bangladesh: incidence, determinants and impact on children's schooling. 2004.	49
43.	Aktar S, Sachu MK, Ali ME. The impact of rewards on employee performance in commercial banks of Bangladesh: an empirical study. Journal of Business and Management. 2012;6(2):9-15.	8
44.	Nigatu D, Gebremariam A, Abera M, Setegn T, Deribe K. Factors associated with women’s autonomy regarding maternal and child health care utilization in Bale Zone: a community based cross-sectional study. BMC women's health. 2014;14(1):79.	9
45.	Victora CG, Wagstaff A, Schellenberg JA, Gwatkin D, Claeson M, Habicht J-P. Applying an equity lens to child health and mortality: more of the same is not enough. The Lancet. 2003;362(9379):233-41.	9
46.	Parveen S. Access of rural women to productive resources in Bangladesh: a pillar for promoting their empowerment. International Journal of Rural Studies. 2008;15(1).	1
47.	Black RE, Allen LH, Bhutta ZA, Caulfield LE, De Onis M, Ezzati M, et al. Maternal and child undernutrition: global and regional exposures and health consequences. The lancet. 2008;371(9608):243-60.	18
48.	Das MB, Amin S, Johnson K, Hossain A. Whispers to voices: Gender and social transformation in Bangladesh. 2008.	170
49.	National Institute of Population Research and Training (NIPORT), Mitra and Associates, ICF International. Bangladesh Demographic And Health Survey 2011. 2013	458

Sl no	Name of the documents	Number of pages
50.	National Institute of Population Research and Training (NIPORT), Mitra and Associates, ICF International. Bangladesh Demographic and Health Survey 2014. Dhaka, Bangladesh: NIPORT, Mitra and Associates, and ICF International, 2016.	354
51.	National Institute of Population Research and Training (NIPORT), Ministry of Health and Family Welfare, ICF. Bangladesh Demographic and Health Survey 2017-18. Dhaka, Bangladesh: NIPORT/ICF, 2020.	511
52.	National Institute of Population Research and Training (NIPORT), Ministry of Health and Family Welfare, ICF. Bangladesh Demographic and Health Survey 2022: Key Indicators Report. Dhaka, Bangladesh: NIPORT/ICF, 2023.	84
53.	UNICEF. Bangladesh. 2021. Available: https://data.unicef.org/country/bgd/. Accessed.	15
54.	Nahar T, Azad K, Aumon BH, Younes L, Shaha S, Kuddus A, et al. Scaling up community mobilisation through women's groups for maternal and neonatal health: experiences from rural Bangladesh. BMC Pregnancy and Childbirth. 2012;12:5.	10
55.	Rahman AE, Hossain AT, Siddique AB, Jabeen S, Chisti MJ, Dockrell DH, et al. Child mortality in Bangladesh - why, when, where and how? A national survey-based analysis. J Glob Health. 2021;11:04052.	14
56.	Rahman AE, Herrera S, Rubayet S, Banik G, Hasan R, Ahsan Z, et al. Managing possible serious bacterial infection of young infants where referral is not possible: Lessons from the early implementation experience in Kushtia District learning laboratory, Bangladesh. PloS one. 2020;15:e0232675.	21
57.	Rubayet S, Shahidullah M, Hossain A, Corbett E, Moran AC, Mannan I, et al. Newborn survival in Bangladesh: a decade of change and future implications. Health Policy and Planning. 2012;27:iii40-iii56.	17
58.	UNICEF. Child survival and the SDGs. 2023. Available: https://data.unicef.org/topic/child-survival/child-survival-sdgs/. Accessed.	2
59.	Gliklich R, Dreyer N, Leavy M. Registries for Evaluating Patient Outcomes: A User's Guide. Two volumes.(Prepared by the Outcome DEcIDE Center [Outcome Sciences, Inc., a Quintiles company] under Contract No. 290 2005 00351 TO7.) AHRQ Publication No. 13 (14)-EHC111. Rockville, MD: Agency for Healthcare Research and Quality. April 2014. Rockville, MD: Agency for Healthcare Research and Quality. 2014.	309
60.	Dokholyan RS, Muhlbaier LH, Falletta JM, Jacobs JP, Shahian D, Haan CK, et al. Regulatory and ethical considerations for linking clinical and administrative databases. American heart journal. 2009;157:971-82.	12
61.	Madhok R, editor Crossing the quality chasm: lessons from health care quality improvement efforts in England. Baylor University Medical Center Proceedings; 2002: Taylor & Francis.	08
62.	WHO, UNICEF. Every newborn: an action plan to end preventable deaths. Geneva: World Health Organization. 2014:2017-18.	58
63.	Mwakyusa S, Wamae A, Wasunna A, Were F, Esamai F, Ogutu B, et al. Implementation of a structured paediatric admission record for district hospitals in Kenya–results of a pilot study. BMC international health and human rights. 2006;6:1-7.	08
64.	Sim J, Wright CC. The kappa statistic in reliability studies: use, interpretation, and sample size requirements. Physical therapy. 2005;85:257-68.	12

Table S2: Key informants

Sl no	Designation	Organization
1.	Programme Manager, National Newborn Health Programme and IMCI	Directorate General of Health Services (DGHS)
2.	Deputy Programme Manager, Newborn Health	Directorate General of Health Services (DGHS)
3.	Deputy Programme Manager, Training and child injury, National Newborn Health Programme and IMCI	Directorate General of Health Services (DGHS)
4.	Deputy Programme Manager, Coordination & Logistics, NNHP & IMCI	Directorate General of Health Services (DGHS)
5.	Deputy Programme Manager, Monitoring & Data Quality, NNHP & IMCI	Directorate General of Health Services (DGHS)
6.	Deputy Programme Manager, Admin & Finance, NNHP & IMCI	Directorate General of Health Services (DGHS)
7.	Senior Director and Senior Scientist, Maternal and Child Health Division	icddr,b
8.	Scientist, Maternal and Child Health Division	icddr,b
9.	Assistant Scientist, Maternal and Child Health Division	icddr,b

Table S3: List of acronyms for the Power matrix mapping figure

Sl no	Type	Acronym	Stakeholder	Power	Interest
1.	National	BPA	Bangladesh Paediatric Association	3	8
2.	National	BNF	Bangladesh Neonatal Federation	2	4
3.	National	BPS	Bangladesh Perinatal Society	3	2
4.	National	DGHS-MNC&AH	Directorate General of Health Services Maternal Newborn Child and Adolescent Health	10	10
5.	National	DGHS-HSM	Directorate General of Health Services-Hospital Services Management	6	9
6.	National	DGHS-NNS	Directorate General of Health Services-National Nutrition Services-Operational Plan	6	6
7.	National	DGHS-CBHC	Directorate General of Health Services Community Based Health Care-Operational Plan	6	2
8.	National	DGFP-MNCRAH	Directorate General of Family Planning Maternal, Child, Reproductive and Adolescent Health	9	2
9.	National	SCI	Save the Children	9	8
10.	National	UNICEF	United Nations International Children’s Emergency Fund	7	9
11.	National	WHO	World Health Organization	8	7
12.	National	PRF	Projahnmo Research Foundation	8	6
13.	National	IPAS	international, non-governmental organization	8	4
14.	National	icddr,b	International Centre for Diarrhoeal Disease Research	9	10
15.	National	UNFPA	United Nations Population Fund.	7	3
16.	District	CS	Civil Surgeon	9	9
17.	District	UH&FPO	Upazila Health and Family Planning Officer	8	9
18.	District	FWV	Family Welfare Visitor	4	5
19.	District	UFPO	Upazila Family Planning Officer	4	3
20.	District	DDFP	Deputy Director Family Planning	7	4
21.	District	SACMO	Sub-Assistant Community Medical Officer-DGFP	3	1
22.	District	RMO	Residential Medical Officer	7	8
23.	District	CONSULTANT	Local Non-Government Organization	6	8
24.	District	NURSE	Local Non-Government Organization	6	6

Table S4: Team members of the Technical Working Committee

Sl no	Designation	Organization
1.	Line Director of MNCAH	DGHS
2.	Programme Manager, National Newborn Health Programme and IMCI	DGHS
3.	Deputy Programme Manager, Newborn Health	DGHS
4.	Deputy Programme Manager, Training and child injury, National Newborn Health Programme and IMCI	DGHS
5.	Deputy Programme Manager, Coordination & Logistics, NNHP & IMCI	DGHS
6.	Deputy Programme Manager, Monitoring & Data Quality, NNHP & IMCI	DGHS
7.	Deputy Programme Manager, Admin & Finance, NNHP & IMCI	DGHS
8.	Medical officer, NNHP & IMCI	DGHS
9.	Deputy Programme Manager, NNS, IPHN	DGHS
10.	Deputy Director of Hospital and Clinics	DGHS
11.	Deputy Programme Manager, Hospital Services Management	DGHS
12.	Deputy Programme Manager, UHC	DGHS
13.	Medical Officer, NNHP & IMCI	DGHS
14.	Focal Point, NBH cell	Save the Children
15.	Advisor (Newborn Health), Government Support, MaMoni-MNCSP	Save the Children
16.	National Consultant-RMNCAH	WHO
17.	MNCH Specialist	UNICEF
18.	Deputy Director	PRF
19.	Senior Director and Senior Scientist, Maternal and Child Health Division	icddr,b
20.	Scientist, Maternal and Child Health Division	icddr,b
21.	Assistant Scientist, Maternal and Child Health Division	icddr,b
22.	Research Investigator	icddr,b
23.	Study Physician	icddr,b
24.	Project Research Physician	icddr,b
